# Mutations in BIN1 Associated with Centronuclear Myopathy Disrupt Membrane Remodeling by Affecting Protein Density and Oligomerization

**DOI:** 10.1371/journal.pone.0093060

**Published:** 2014-04-22

**Authors:** Tingting Wu, Zheng Shi, Tobias Baumgart

**Affiliations:** Department of Chemistry, School of Arts & Sciences, University of Pennsylvania, Philadelphia, Pennsylvania, United States of America; Institut Curie, France

## Abstract

The regulation of membrane shapes is central to many cellular phenomena. Bin/Amphiphysin/Rvs (BAR) domain-containing proteins are key players for membrane remodeling during endocytosis, cell migration, and endosomal sorting. BIN1, which contains an N-BAR domain, is assumed to be essential for biogenesis of plasma membrane invaginations (T-tubules) in muscle tissues. Three mutations, K35N, D151N and R154Q, have been discovered so far in the BAR domain of BIN1 in patients with centronuclear myopathy (CNM), where impaired organization of T-tubules has been reported. However, molecular mechanisms behind this malfunction have remained elusive. None of the BIN1 disease mutants displayed a significantly compromised curvature sensing ability. However, two mutants showed impaired membrane tubulation both *in vivo* and *in vitro*, and displayed characteristically different behaviors. R154Q generated smaller membrane curvature compared to WT N-BAR. Quantification of protein density on membranes revealed a lower membrane-bound density for R154Q compared to WT and the other mutants, which appeared to be the primary reason for the observation of impaired deformation capacity. The D151N mutant was unable to tubulate liposomes under certain experimental conditions. At medium protein concentrations we found ‘budding’ structures on liposomes that we hypothesized to be intermediates during the tubulation process except for the D151N mutant. Chemical crosslinking assays suggested that the D151N mutation impaired protein oligomerization upon membrane binding. Although we found an insignificant difference between WT and K35N N-BAR in *in vitro* assays, depolymerizing actin in live cells allowed tubulation of plasma membranes through the K35N mutant. Our results provide insights into the membrane-involved pathophysiological mechanisms leading to human disease.

## Introduction

Cell membranes display a wide variety of shapes, including flat sheets, vesicles and tubules. Dynamic membrane remodeling occurs during phenomena such as membrane trafficking, organelle biogenesis, and cell division. Changes in membrane morphology can be accomplished through translocation and assembly of membrane sculpting proteins[1]–[5].

Amphiphysin II, also called BIN1, is such a membrane deforming protein. It was first identified as a tumor repressor by its interaction with MYC oncoproteins. In accordance with that role, BIN1 expression was found to be reduced in cancer cell lines[6]–[8].

The human BIN1 gene is subject to alternative splicing in a cell-type-specific manner[9]–[11]. Isoform 8 is primarily expressed in striated muscle tissues. This isoform contains a phosphatidylinositol-4,5-bisphosphate (PI(4,5)P_2_) binding sequence encoded by exon10[12], [13]. In skeletal myocytes, BIN1 locates on tubular membrane invaginations called transverse tubules (T-tubules). T-tubules incorporate Ca^2+^ releasing channels and ryanodine receptors, and are a membranous platform critical for synchronous Ca^2+^ release[14]. Acute knockdown studies of BIN1 in skeletal muscle have revealed disorganized T-tubule formation and impaired intracellular Ca^2+^ signaling[15]. Additionally, BIN1 is required for C2C12 myoblast fusion and differentiation[13], [16].

**Figure 1:**
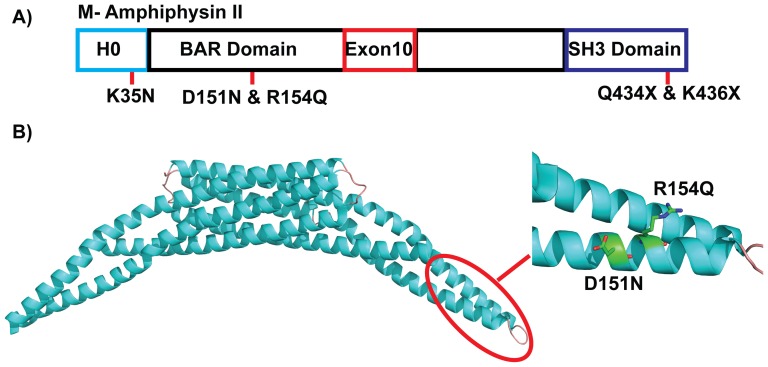
Location of CNM-associated mutations in BIN1 protein. A) Schematic location of K35N, D151N, R154Q, and two nonsense mutation in SH3 domain found in CNM patients in human BIN1 protein. B) Cartoon structure of human BIN1 N-BAR domain (PDB ID code: 2FIC). Residues D151 and R154 are shown as sticks at the distal arm of a BAR domain dimer.

Full-length BIN1 isoform 8 contains an N-terminal BAR domain, a Myc-binding domain, and a C-terminal Src homology 3 domain (SH3 domain) (Fig. 1A)[13], [17]. BIN1 associates with membranes peripherally through its N-terminal BAR domain and binds dynamin2 via its SH3 domain[17]. The crystal structure of a dimeric BAR domain shows a six-helix bundle core with two arms that form a crescent shape (Fig. 1B)[18]. Positively charged amino acids are located at the concave surface that forms the membrane binding interface[1]–[3], [19]. Although not resolved in the crystal structure, residues 1–36 are predicted to fold into an amphipathic helix upon membrane binding[17]. This helix inserts into the membrane leaflet and thereby facilitates curvature generation[4], [8], [20].

Multiple mechanisms have been proposed for how N-BAR domains induce membrane curvature. These include 1) scaffolding by imposing the intrinsic curvature of a BAR dimer to bend the membrane; 2) hydrophobic insertion of amphipathic helix causing asymmetry between two leaflets; 3) protein oligomerization and lattice formation to stabilize curved membranes; and 4) recently discovered protein crowding effects leading to membrane tubulation[21]–[23].

Interestingly, several mutations have been identified in the BIN1 gene in patients with autosomal recessive centronuclear myopathy (CNM). CNM is an inherited neuromuscular disorder characterized by muscle weakness, abnormal localization of nuclei, and growth retardation[24]–[26]. To date, five mutations in the BIN1 gene have been discovered in CNM. Among these, three mutations are in the N-BAR domain region: K35N, D151N, and R154Q. The other two are nonsense mutations in the C-terminal SH3 domain: Q434X and K436X (numbering for human isoform 8, Fig. 1A)[17], [27]–[29]. These mutations in the N-BAR domain of BIN1 result in defective T-tubule biogenesis in skeletal myocytes. The nonsense mutations in the SH3 domain do not compromise the membrane tubulation ability of BIN1, but abolish recruitment of dynamin2 on tubules[17], [30], [31]. Disrupted membrane remodeling by BIN1 mutations is thus clearly connected with centronuclear myopathy, but the molecular mechanism underlying the effects of each point mutation in BIN1 on membrane interactions is poorly understood.

In this study, we focused on three CNM-related mutations in the BIN1 N-BAR domain and investigated differences among BIN1 N-BAR variants in the context of membrane association and curvature generation. Our *in vivo* cellular studies of BIN1 N-BAR variants confirmed that all three mutations in the BIN1 N-BAR domain impaired membrane tubulation. We demonstrated that lipid composition played an important role in regulating tubulation abilities among BIN1 N-BAR mutants in liposome tubulation assays. Both the D151N and R154Q mutations in the BIN1 N-BAR domain led to significant reduction in tubulation for lipid compositions mimicking physiological conditions. We then studied protein densities on giant unilamellar vesicles (GUVs) and found that the R154Q mutation caused reduced protein binding to the membrane. However, interestingly the D151N mutant retained a similar curvature sensing ability on pulled tethers as WT N-BAR and was also able to reduce equilibrium pulling force on membrane tethers relative to bare membranes. Chemical crosslinking and protein titration in tubulation assays suggested that D151N was deficient in protein oligomerization on membranes. Strikingly, we failed to identify a significant deviation of recombinant K35N N-BAR from WT in *in vitro* assays. This implies that the K35N mutation does not disrupt N-terminal helix hydrophobic insertion into the lipid membrane, which is a widely accepted mechanism for the N-BAR domain protein in curvature generation[4]. Lastly, we provided preliminary evidence that the cytoskeleton regulates membrane tubulation activity of BIN1 in cells.

## Results and Discussion

### Point mutations associated with CNM in BIN1 impair membrane remodeling

BIN1, as a BAR domain containing protein, is implicated in membrane remodeling during T-tubule generation. Previous research has shown that overexpression of BIN1 in cells leads to membrane tubulation. The first two N-BAR domain mutations (K35N and D151N) found in CNM patients were reported to abolish the membrane deforming function of BIN1 in COS-1 cells. However, for the most recently discovered mutant (R154Q), only tissue biopsy data are available thus far. These indicate that R154Q also disrupts T-tubule organization and thus triad function at sarcoplasmic reticulum (SR)/T-tubule junctions [28], [29].

In order to test the hypothesis that all disease mutations found in the BIN1 BAR domain impair membrane tubule formation, we transiently transfected C2C12 myoblasts with BIN1 N-BAR* wild type (WT) and CNM-related mutants conjugated with mKate at the C-terminus. The N-BAR* domain is distinguished from the N-BAR domain by a longer sequence that includes exon10 (Fig. 1A). We used the BIN1 N-BAR* domain because it has been reported that the polybasic sequence coded by exon10 is required for targeting to the plasma membrane mediated by PI(4,5)P_2_ and that *in vivo* membrane tubulation is not observed by BIN1 N-BAR domains[13].

**Figure 2:**
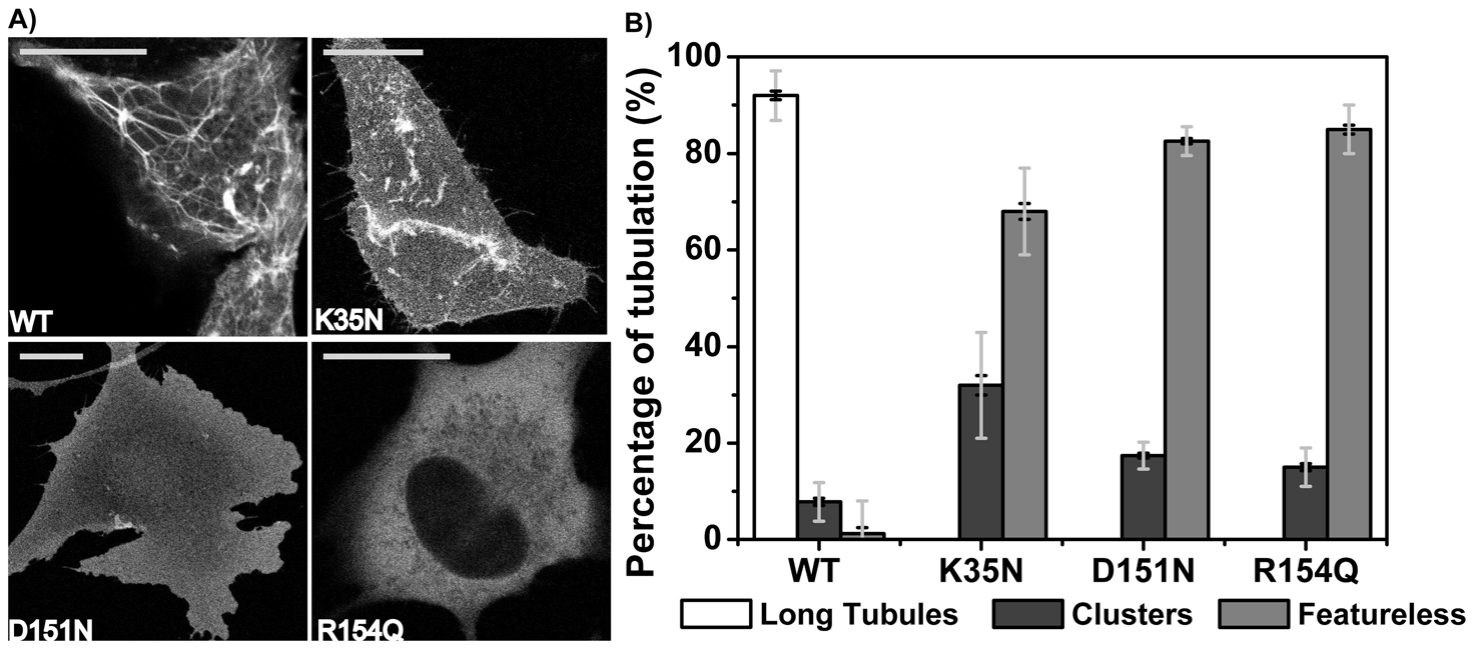
Expressing CNM-related mutants in C2C12 cells leads to loss of membrane tubulation. A) BIN N-BAR* WT and mutants are fused to mKate at C-terminus and transiently transfected in C2C12 myoblasts. Cells are imaged by confocal fluorescence microscopy. Scale bar: 20 µm. B) Quantification of three types of membrane deformations by BIN1 N-BAR* variants. Clusters are defined as structures with lengths not longer than five times of their width. Over 50 cells were analyzed in each separated experiments. Error bars: standard error of the mean in black and standard deviation in light grey. Student *t*-test for statistical significance: n.s: p>0.05, *: p<0.05, **: p<0.01, ***: p<0.001.

Confocal micrographs in Fig. 2A demonstrated that BIN1 N-BAR* WT expression resulted in the generation of invaginated membrane tubules, while cells expressing the CNM-associated mutant proteins showed essentially homogeneous cytosolic fluorescence. To exclude potential membrane morphological influences by the fluorescence tags, mKate (Fig. S1A) or EGFP (Fig. S1B) alone were expressed in C2C12 myoblasts. All cells were found to express fluorescence proteins (mKate or EGFP) in a homogeneous manner. To quantitatively compare membrane deforming differences, we categorized tubules in live cells into three groups based on tubule lengths and calculated the percentages of cells in each group[17]. As shown in Fig. 2B, on average, over 90% of cells with over-expressed WT N-BAR* had long tubules generated, while this phenotype was absent in all disease mutants. In cells transfected with disease mutants, we found fluorescent clusters in 30% of the cells. These clusters may be protein aggregates or short tubules that we were not able to distinguish due to the optical resolution limit.

**Figure 3:**
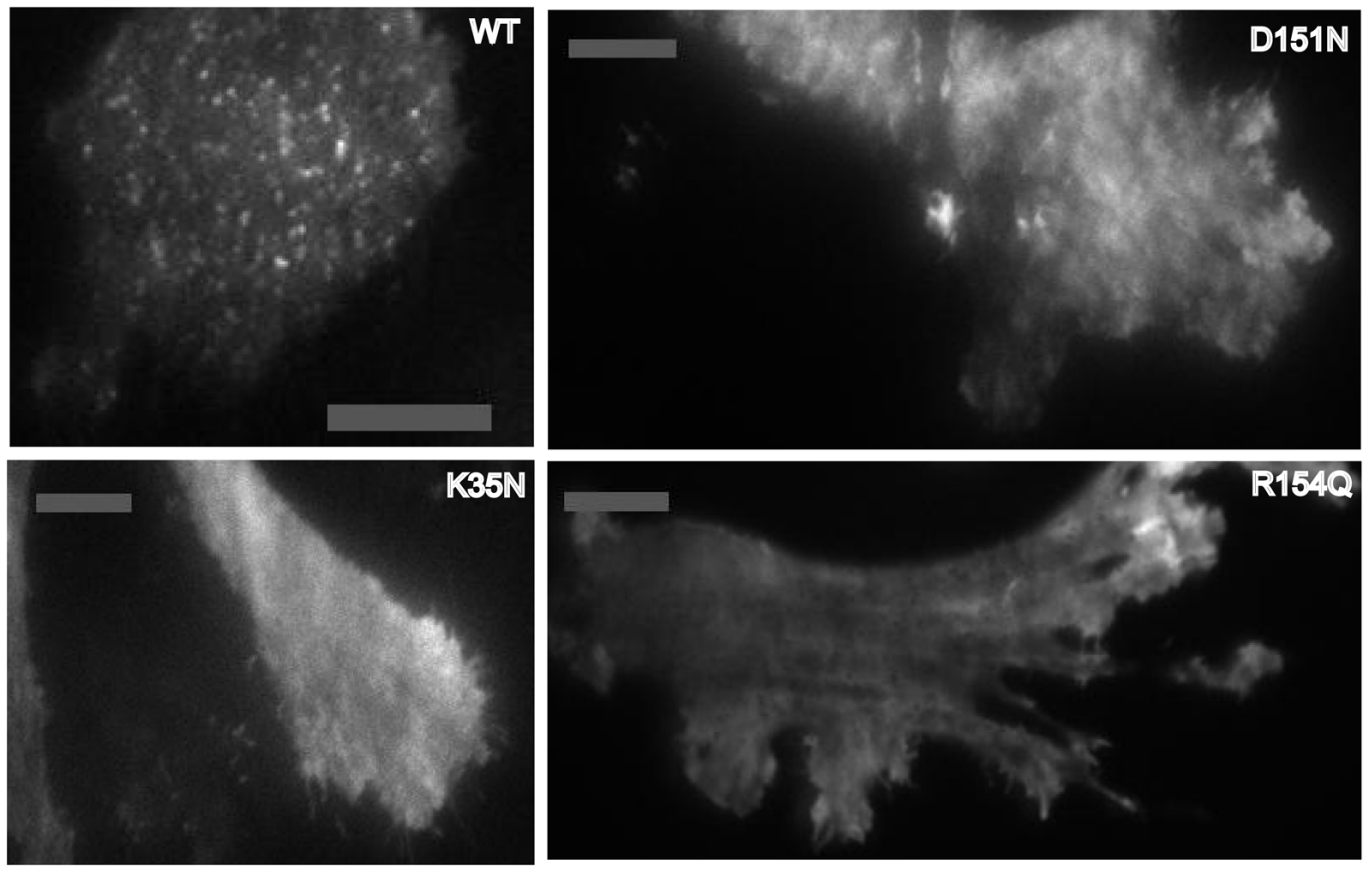
TIRF micrographs show homogeneous fluorescence of CNM-related mutants on plasma membrane. BIN N-BAR* WT and mutants are fused to EGFP at N-terminus and transiently transfected in C2C12 myoblasts. Cells are imaged by TIRF fluorescence microscopy. Scale bar: 20 µm. All variants of BIN1-N-BAR* proteins show binding ability on plasma membrane. However, only WT BIN1-N-BAR* generates fluorescent puncta on the membrane.

To understand the origins of membrane tubule generation, we used Total Internal Reflection Fluorescence microscopy (TIRF) to examine the spatial organization on the plasma membrane of BIN1 N-BAR* domains conjugated with green fluorescent protein (EGFP). The introduction of fluorescent tags at the N-terminus might affect N-terminal helix membrane insertion. However, we did not observe changes in *in-vivo* tubulation phenotypes among BIN1 N-BAR* variants when proteins were either N-terminally (Fig. S2) or C-terminally (Fig. 1) labeled. Consistently, distinct fluorescent speckles were only present on plasma membranes of cells transfected with N-BAR* WT, whereas disease mutants were distributed more evenly on the membrane (Fig. 3).

From the TIRF imaging results we conclude that: 1) most of the membrane tubules induced by BIN1 N-BAR* are oriented perpendicularly to the TIRF plane. Thus, fluorescence projections on the plasma membrane show up as speckles; 2) only the WT protein, which possesses the capacity for cellular membrane tubulation, shows significant clusters at the plasma membrane.

The findings from this section emphasize that BIN1 N-BAR* acts as a membrane curvature generator, causing membrane tubulation in cells, and that this function is perturbed by disease mutations.

### *In vitro* tubulation abilities of recombinant N-BAR domain variants vary with lipid composition

To further characterize the BIN1 N-BAR domain and its mutants, we purified WT BIN1 N-BAR domains as well as disease mutants. Because we neither observed retention volume shifts in size exclusion chromatography, nor any changes in CD spectra, we believe that none of the disease mutations had significant effects on the folding of the protein (data not shown).

**Figure 4:**
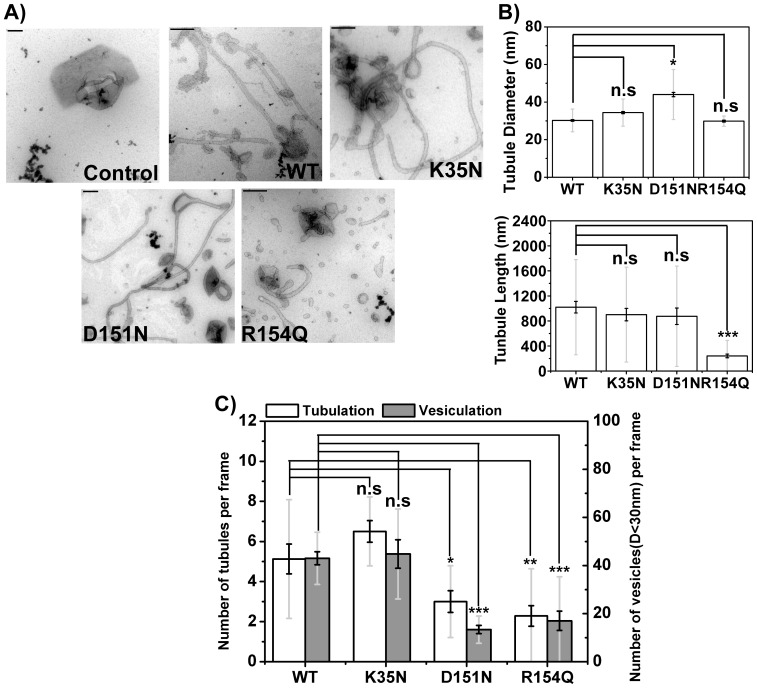
On membrane with high negative charge, only R154Q shows mild reduction in tubulation. A) Electron micrographs of liposomes (100% DOPS) tubulated by BIN1 N-BAR and mutants. Membrane tubules are observed for all BIN1 N-BAR variants. Scale bar: 200 nm. B) Quantifications of tubule diameter and length by BIN1 variants. R154Q mutation leads to increased tubule diameters and decreased tubule length comparing to WT BIN1 protein. C) Averaged occurrence of membrane tubules and vesiculated liposomes in each micrograph. K35N and D151N mutant are similar to WT N-BAR in membrane tubulation. All the disease mutants cause decrease in vesiculation to varied extent. Over 30 images were analyzed for quantifications. Error bars: standard error of the mean in black and standard deviation in light grey. Student *t*-test for statistical significance: n.s: p>0.05, *: p<0.05, **: p<0.01, ***: p<0.001.

To characterize curvature generation by BIN1 N-BAR domains and its mutants, we adopted an *in vitro* liposome deformation assay involving negative staining transmission electron microscopy (TEM). Negative staining is a widely accepted method to study biological macromolecules. It involves embedding liposome-protein complexes adsorbed to sample grids in a dried heavy metal solution for contrast enhancement[32]. We first used large unilamellar liposomes (LUVs) composed of 100% DOPS to maximize electrostatic interactions between protein and membrane without the potential complications of lipid demixing. Contrary to the observations from cellular studies described above, tubules were found in all the samples when BIN1 variants were incubated with liposomes composed of 100% DOPS (Fig. 4A). We used tubule diameter and tubule length as two parameters to quantify the strength of the membrane shaping ability of BIN1 N-BAR domains. Shown in Fig. 4B, the averaged tubular diameter induced by BIN1 N-BAR WT was 34±1 nm (note that here and below uncertainties are expressed as standard errors of the mean if not indicated otherwise), which is comparable to the reported tubule dimensions generated by N-BAR domain proteins (ranging from 20–50 nm)[20], [33]. The length of tubules varied from 300 nm to 4 µm with an averaged tubule length around 549±43 nm. No significant differences between WT and K35N were found with respect to either tubule diameter or length (K35N: 35±1 nm in diameter and 436±22 nm in length). For D151N, although there was a slight increase in the tubule diameter (40±1 nm), the averaged tubule length was similar as WT (D151N: 385±26 nm). For the R154Q mutant, tubules were shorter (190±11 nm, 3 fold decreased) suggesting reduced tubulation ability of R154Q. We acknowledge that dehydration during sample preparation may cause variations in geometric measurements that can be avoided using the alternative method of Cryo-EM imaging[32], [34]. The diameters of the tubules generated by endophilin N-BAR domain ranged from 24 nm to 28 nm in Cyro-EM studies. The tubule dimensions we measured here were slightly higher than the published data, which could result either from tubule collapse during the drying process, variations in the lipid composition, or protein concentration used in different studies[35], [36].

Two types of membrane deforming events occur as consequences of N-BAR protein binding: tubulation and vesiculation. N-BAR domains are known to be able to deform large liposomes into vesicles with diameters smaller than 30–40 nm[37]. Therefore, we quantified both tubulation and vesiculation events as a measure of membrane deformation ability of peripheral proteins.

We compared the average number of tubulation and vesiculation events in 30 individual image fields. WT and K35N had similar deformation capacities with 5–6 tubules and 48 vesiculated liposomes (diameter was less than 30 nm) on average per frame (Fig. 4C). Tubule numbers for D151N and R154Q were reduced to 71±12% and 40±6% of that for WT. Vesiculation numbers for D151N and R154Q, however, decreased more significantly to 22±5% and 27±4% of the vesiculation events observed for WT protein.

To conclude, on 100% DOPS membranes, BIN1 N-BAR domains are able to deform spherical membranes into tubules or small vesicles. Mild decrease in the membrane deformation capacity is found in the D151N and R154Q mutants.

Because with model membranes consisting of 100% DOPS we found only mild impairment of deformation capacity of disease mutants (Fig. 4) compared to the significant differences observed in cells (Fig. 2), we next tested the hypothesis that lipid composition plays a role in regulating deformation capacity of BIN1 variants. We chose a lipid composition meant to better mimic the headgroup composition of the inner plasma membrane leaflet: DOPC:DOPS:DOPE:PI(4,5)P_2_ = 6∶2∶1∶1. Here, DOPS and PI(4,5)P_2_, which are enriched in the inner leaflet of the plasma membrane[38], provide negative charge promoting electrostatic interactions with the BAR domain while the presence of the smaller headgroup in DOPE lipids results in more lipid packing defects that facilitate insertion of amphipathic helices leading to enhanced tubulation and vesiculation[39]–[41]. In this composition, the overall negative charge density on the membrane decreases to 50% rather than 100% as a result of the reduced DOPS percentage and −3 charge per PI(4,5)P_2_ molecule at physiological conditions[42], [43]. We note that PI(4,5)P_2_, as a multivalent lipid, could introduce specific effects on protein-membrane interactions[44]. Despite this potential complication we aim, with this more physiological lipid composition, to provide biologically relevant insights into how BIN1 N-BAR membrane interactions differ among our mutants.

**Figure 5:**
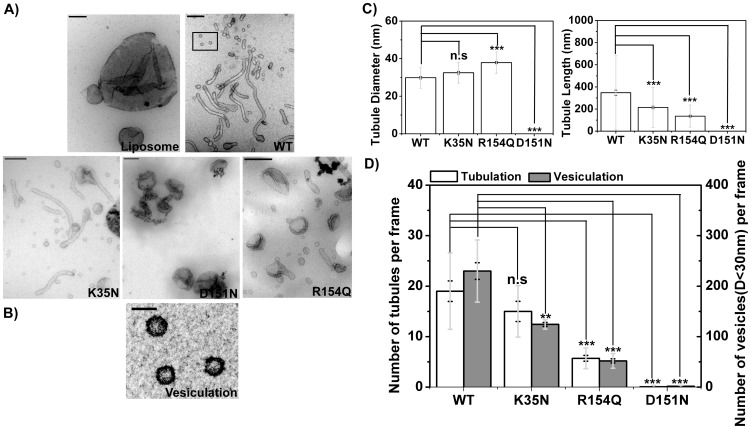
Lowering membrane negative charge further reduces tubulation ability for the D151N and R154Q mutants. A) Electron micrographs of liposomes (60% DOPC/20% DOPS/10% DOPE/10% PI(4,5)P_2_) incubated with BIN1 N-BAR and the mutants. WT and K35N still strongly deform vesicles. Short tubules are observed in R154Q samples. Almost complete loss of curvature generation is observed in D151N. Scale bar: 200 nm. Electron micrograph in B) is the zoom-in image boxed in A). Uniform small vesicles with 28±7 nm diameters are found after incubation with N-BAR proteins. Scale bar: 50 nm. C) Quantifications of tubule diameter and length by BIN1 variants. R154Q mutation shows compromised curvature generation. D) Quantification of the averaged occurrence of membrane tubules and vesiculated liposomes in each micrograph. K35N shows similar number of tubules per frame and mild decrease in vesiculation. Both D151N and R154Q lead to significant decrease in tubulation and vesiculation. Over 30 images were analyzed for quantifications. Error bars: standard error of the mean in black and standard deviation in light grey. Student *t*-test for statistical significance: n.s: p>0.05, *: p<0.05, **: p<0.01, ***: p<0.001.

For this lipid composition, electron micrographs of liposomes incubated with BIN1 N-BAR variants are shown in Fig. 5A. WT BIN1 N-BAR protein acted as a strong curvature generator. The averaged tubule diameter was 29±1 nm with a tubule length distribution ranging from 200 nm to 2 µm. With this lipid composition, more vesiculated liposomes (with diameters less than 30 nm) were found on grids (Fig. 5B) than for the 100% DOPS composition. Compared to WT BIN1, membrane tubules induced by K35N had similar width, but tubule lengths reduced to 61±5% of those generated by WT. The R154Q mutant showed wider (37±1 nm) and shorter (135±9 nm) tubules. Strikingly, we found severely impaired membrane deformation for D151N (Fig. 5C): Among over 30 analyzed EM micrographs, no tubulation or vesiculation was observed (Fig. 5A&D). Differences among N-BAR variants are clearly demonstrated in the tubulation and vesiculation analyses in Fig. 5D. While K35N showed a number of tubulation events per frame that was comparable to the WT protein, the other mutants exhibited decreased probabilities of tubulation and vesiculation from liposomes.

### R154Q causes compromised membrane association density

Our observations from the tubulation assays suggest that different disease mutations have varying effects on membrane deformation capacity. Membrane association is the first step required for membrane curvature sensing and generation. It has been shown that curvature-coupling is protein density dependent[45]. Here we used a GUV binding assay to quantify protein density to probe if point mutations in the BIN1 BAR domain impair membrane binding.

**Figure 6:**
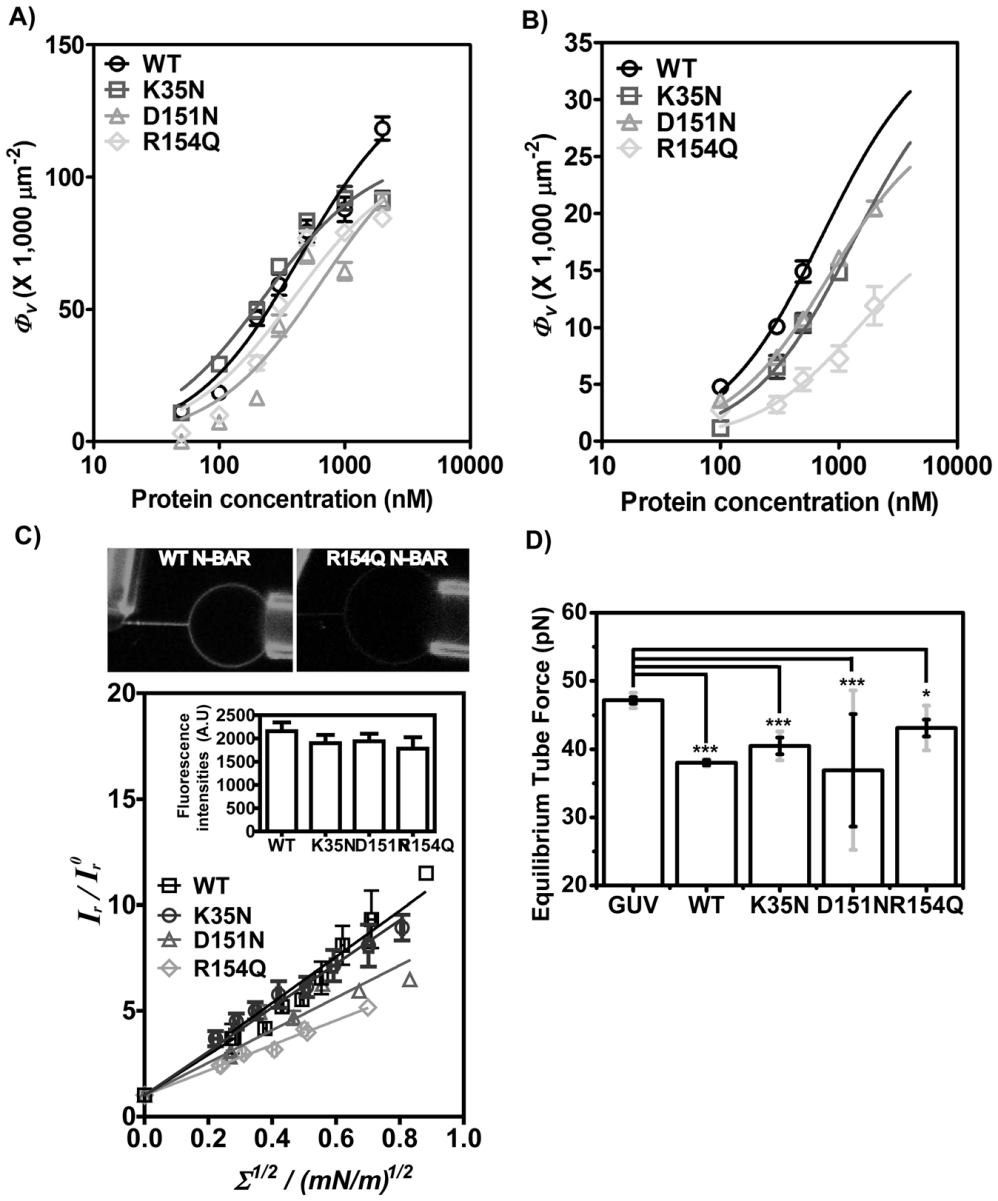
R154Q mutation lowers membrane association density. Protein adsorption isotherm on GUVs composed of A) 100% DOPS and B) 60% DOPC, 20% DOPS, 10% DOPE, and 10% PI(4,5)P_2_. Protein and vesicles are incubated in 20 mM Hepes, 50 mM NaCl, 1 mM DTT, pH 7.4 buffer at room temperature for 30 minutes before imaging on confocal fluorescence microscopy. Green fluorescence intensities on vesicle equators are measured and normalized to the total membrane area. Absolute molecule density is calculated according to the calibration curve. Data are fitted by the Langmuir isotherm model to obtain *K_d_* and *B_max_*. Membrane bound density of R154Q dramatically decreases on biomimetic membrane. The error bars are the standard error of the mean of 10 measured GUVs. C) Quantitative analysis of the curvature sorting abilities of BIN1 N-BAR domain and its mutants. A membrane tether is pulled from a micropipette-aspirated GUV (60% DOPC, 20% DOPS, 10% DOPE, 10% PI(4,5)P_2_, labeled by Texas-Red lipid dye) by a polystyrene bead and imaged by confocal fluorescence microscopy. At 100 nM, WT N-BAR fluorescence on GUV and tether is brighter than R154Q N-BAR as shown in the upper panel. WT (100 nM), K35N (100 nM), D151N (100 nM) and R154Q (600 nM) are pre-incubated with GUVs in 20 mM Hepes, 50 mM NaCl, 1 mM DTT, pH 7.4 buffer at room temperature for 30 minutes before transfer to aspiration chamber. By increasing membrane tension controlled by aspiration pressure, protein partitioning on tubular membrane tether and decrease in lipid fluorescence on tether is observed. Curvature coupling parameter 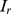 */* 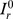 from images recorded by Kalman-averaged confocal xz line-scan images is plotted with square root of membrane tension, revealing a linear relationship. Data from six vesicles was binned (vertical error bars represent standard error of the mean of 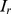 */* 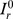 and horizontal error bars show standard error of the mean of square root of tension). Inset graph demonstrates similar fluorescence intensity on GUVs analyzed for four BIN1 variants. Error bars are standard errors of the mean. D) Equilibrium force of tethers (pulled from GUVs with composition (60% DOPC, 20% DOPS, 10% DOPE, 10% PI(4,5)P_2_) measured by optical trap at membrane tension of 0.21±0.003 mN/m. Error bars: standard error of the mean in black and standard deviation in light grey. Student *t*-test for statistical significance: n.s: p>0.05, *: p<0.05, **: p<0.01, ***: p<0.001.

In Fig. 6A&B, we quantified BIN1 N-BAR membrane binding density on giant unilamellar vesicles using the two lipid compositions described above as a function of increasing bulk protein concentration in buffer containing 50 mM NaCl. In these binding studies, care was taken to maintain protein solution concentration at values low enough to prevent microscopically visible tubulation. Recombinant BIN1 N-BAR variants were labeled with Alexa-488 on endogenous cysteines and we then measured mean fluorescence intensities per pixel along the GUV equator. Fluorescence signals were converted to protein membrane binding densities based on an adapted calibration method [45]–[47].

On the 100% DOPS vesicles, the density of membrane-bound protein increased with increasing protein solution concentration, as expected. The membrane binding affinities (*K_d_*) that we obtained were similar among BIN1 N-BAR variants. The membrane binding dissociation constants *K_d_* are: 320 nM±51 nM (WT), 400 nM±29 nM (K35N), 540 nM±172 nM (D151N) and 640 nM±94 nM (R154Q). From these affinities, one finds that the maximal binding free energy difference comparing WT and mutants is only about 0.7 *k_B_T*, i.e. on the order of thermal fluctuations. The saturation densities for the disease mutants were only slightly lower compared to the WT N-BAR domain (Fig. 6A). The overall high protein packing densities (>100,000 per μm^2^), even for disease mutants on 100% DOPS membranes, are consistent with the notion that the mutants are still able to generate tubules from liposomes but with shorter lengths and lower probabilities, as shown in Fig. 4, because of the slightly decreased protein densities compared to WT.

For the composition 60%DOPC/20%DOPS/10%DOPE/10%PI(4,5)P_2_ (Fig. 6B), where disease mutants show significant variation in tubulation abilities, the membrane dissociation constants increased due to the reduced membrane charge. The observed membrane dissociation constants *K_d_* are: 720 nM±173 nM (WT), 1300 nM±346 nM(K35N), 1000 nM±415 nM (D151N) and 1800 nM±57 nM (R154Q). The differences among *K_d_* values are on the order of thermal fluctuations. However, we obtained the saturation protein densities *B_max_* from the curve fitting and found that *B_max_* of the disease mutants were reduced compared to WT N-BAR, especially for the R154Q mutant. *B_max_* of the R154Q mutant was 54±7% of the saturation density for WT N-BAR domain on the membrane. The decreases in *B_max_* for the K35N (95±15%) and D151N (80±7%) mutants were less noticeable. These data suggest that R154Q impairs membrane association more severely compared to the other N-BAR variants considered here.

Recent studies have assigned an important role of protein density on membranes in influencing curvature-coupling. Even protein crowding alone can lead to membrane tubulation[23], [45]. Our results from protein density quantification on GUVs suggest that the R154Q mutation reduces protein packing density on the membrane due to the loss of a positively charged residue at position 154, thus reducing electrostatic interactions between the N-BAR domain and negatively-charged membrane. Arginine is also often found inserted into the lipid bilayer[48]. Mutational studies of peptide-membrane interactions have revealed that arginine density governs the strength of peptide-lipid headgroup interactions and depth of insertion into the lipid membrane[49]. The crystal structure of the BIN1 N-BAR domain suggests that residue 154 is not oriented towards the membrane. However, our results are consistent with the hypothesis that R154 contributes to membrane binding.

N-BAR domains are known to function as curvature sensors[21]. The SLiC (**S**ingle **Li**posomes of different diameters and therefore **C**urvature) assay has revealed that N-BAR domains of endophilin preferentially bind to smaller liposomes with higher affinity and density[50], mainly through membrane defect sensing ability via the amphipathic H_0_ helix[51]. It has also been shown before that the N-BAR domain from endophilin is able to partition onto highly curved membranes[52]. We next asked the question if compromised curvature sensing ability caused the impaired membrane deformation capacity of the D151N mutant.

To investigate the ability of the disease mutants to sense membrane curvature, we used a tether-pulling system to quantify the protein partitioning ratio between flat and curved membranes. In this assay, a tubular membrane was pulled from a GUV (composed of 59% DOPC/20% DOPS/10% DOPE/10% PI(4,5)P_2_/0.5% DSPE-Bio-PEG2000/0.5% Texas Red-DHPE) by a polystyrene bead aspirated by a micro-pipette (for fluorescence imaging) or trapped by optical tweezers (for mechanical force measurement on tubule)[52]–[54]. A typical fluorescence image of a tether-pulled vesicle is shown in Fig. 6C (top panel). At a membrane tension of 0.12 mN/m, increased fluorescence signal on the tubular membrane relative to the quasiflat GUV was observed in WT N-BAR at 100 nM concentration. In contrast, significantly reduced fluorescence was found on vesicles incubated with R154Q under the same conditions, confirming our conclusion that R154Q binds to membranes more weakly than the WT protein.

With increasing membrane tension (regulated through increasing pipette aspiration pressure), protein fluorescence signals on tethers were observed to increase. The curvature partitioning 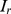 */* 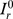 is defined as a ratio of fluorescence signals on the tether (superscript *t*) from protein (green) and lipid (red) normalized to a corresponding ratio on the GUV (superscript *v*), 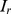 */* 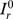 = (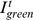 */* 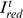)/(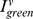 */* 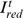).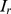 */* 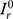 is observed to vary linearly with the square root of membrane tension; *Σ^1/2^* (Fig. 6C), in accordance with simple first-order thermodynamic theories[21], [52]–[56], and similar to what has been reported for ENTH and other types of N-BAR domains[45], [53]. We note that curvature sorting behavior is known to be protein density dependent[45]. Therefore, in the experiments just described, only vesicles showing comparable fluorescence intensities on GUVs were analyzed, as shown in the inset in Fig. 6C. An *F*-test of the linear regressions revealed that the slopes of the curvature sorting/square root of tension relationship comparing WT and K35N are indistinguishable. Although 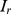 */* 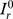 values for the D151N and R154Q mutants are slightly lower compared to WT N-BAR, these two mutants both show that the curvature sorting ratios 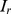 */* 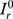 increase proportionally to the square root of membrane tension. The slopes of the fluorescence intensity ratio for all the BIN1 variants investigated here deviate significantly from the null hypothesis of absent curvature sorting (slope  = 0). Results from the curvature sorting assay imply that CNM mutations do not eliminate the curvature sensing ability of N-BAR domains. The defective membrane deformation ability, especially for the D151N mutant, cannot be attributed to the loss of membrane curvature sensing ability.

We next aimed to characterize curvature generation capacities of BIN1 N-BAR domains in more detail. The measurement of membrane tether pulling forces is a powerful assay to probe the mechanical influence of protein binding to membranes. With the help of optical tweezers, forces needed to mechanically stabilize tethers can be monitored in real time[54]. As expected, the presence (100 nM bulk concentration) of BIN1 N-BAR domains was observed to reduce tether pulling forces relative to a pure lipid control at a membrane tension of 0.21±0.003 mN/m (Fig. 6D). This observation implies that binding of BIN1 N-BAR domains on the tether stabilizes membrane curvature.

At the same bulk protein concentrations as the wt protein, equilibrium pulling forces on the tethers covered by BIN1 N-BAR mutants were lower and significantly different from the ones on a bare lipid tube, based on a Student *t* test. Although the R154Q mutant showed a higher pulling force on average compared to the other mutants, the differences among the mutants are not statistically significant. This is consistent with the observation that we found similar curvature-coupled protein sorting behaviors among the BIN1 variants. Particularly, the D151N mutant is able to sense and stabilize the pulled membrane tethers, but it is *not* able to induce spontaneous tubule formation from liposomes (Fig. 5). A decrease of the curvature generation capacity of the R154Q mutant relative to the other mutants and the wt protein is likely to be caused by the decreased membrane association density (Fig. 6B).

To summarize, membrane curvature sorting is displayed by all CNM-associated mutant proteins, and they are able to stabilize cylindrical membrane curvatures. The significantly higher pulling force measured on GUVs incubated with the R154Q mutant is a result of reduced membrane-bound density.

### D151N is unable to initiate curvature required for tubule growth, possibly due to impaired protein assembly/oligomerization on the membrane

As shown above, the D151N mutant binds membranes and senses membrane curvature equivalently to the WT protein. However, the tubulation assay clearly demonstrated that the D151N mutation can impair tubulation under certain conditions.

During clathrin-mediated endocytosis (CME), proteins including clathrin, AP2 and FCHo1/2, arrive at the plasma membrane at an early stage and nucleate to form initial buds that recruit other proteins, including BAR domain proteins, to further promote membrane invagination and scission[57]–[61]. In our tubulation assay, the initiation of membrane tubulation occurs via BAR domain protein binding only.

**Figure 7:**
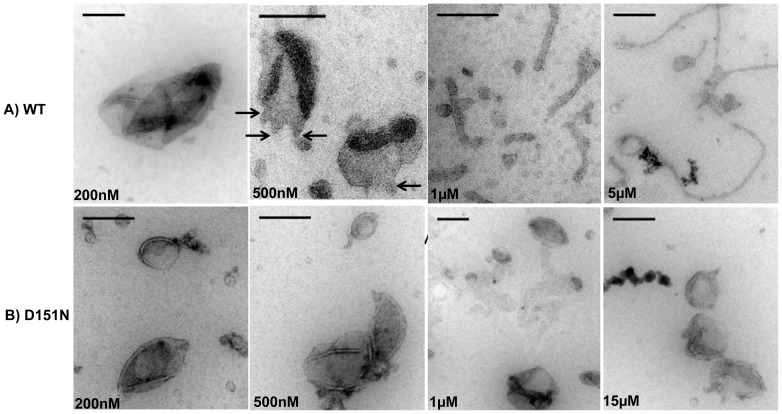
Spontaneous tubulation by BIN1 N-BAR includes ‘membrane budding’ step which is compromised in the D151N mutant. A) Electron micrographs of liposome tubulation by BIN1 N-BAR WT at protein concentrations ranging from 200 nM to 5 µM. Scale bar: 200 nm. At low protein concentration, no morphology deformation is observed. At 500 nM, membrane showed waviness at liposome edges. Further increasing protein concentration results in the growth of longer membrane tubules. B) Titrating D151N N-BAR in tubulation assay (200 nM–15 µM) does not change liposome morphologies.

In order to test the possibility that D151N is deficient in membrane budding initiation, we varied protein concentration in the EM tubulation assay to monitor membrane morphology changes as a function of protein concentration. We first examined BIN1 N-BAR WT in this manner. Fig. 7A shows micrographs of liposomes incubated with BIN1 N-BAR WT at various protein concentrations. At a low protein concentration of 200 nM, the morphologies of liposomes were similar to a lipid control (Fig. 5A). When increasing protein concentration to 500 nM, membrane ‘wobbles’ appeared, as indicated by the arrows in Fig. 7A. We speculate that those buds serve as sites for tubule growth through recruitment of additional proteins. Consistent with that idea, when we further raised protein concentration to 1 µM and 5 µM, tubule generation set in and tubule diameters decreased with increasing protein concentration (Fig. 7A). However, we failed to observe membrane morphology changes for the D151N mutant (Fig. 7B) for a wide range of protein concentrations (200 nM-15 µM). Because we have shown above that the ability to reduce the pulling force of a cylindrical membrane tether, as well as curvature sensing, are *not* eliminated by D151N, we hypothesize that D151N impairs membrane budding at the onset of the tubulation process.

Thus far, our results suggest that the D151N mutation interferes with membrane curvature initiation only, but retained the capacity to stabilize cylindrical membrane curvature (Fig. 6C&D). We next asked, if the addition of a small amount of WT protein (large enough to cause budding (Fig. 7A) but small enough to prevent significant tubulation) might rescue the curvature initiation defect observed in the presence of D151N only. When only 500 nM WT N-BAR was incubated with liposomes, vesicle boundary wobbling (Fig. 7A) was observed, in addition to the generation of some short tubes (Fig. S3B). On the other hand, 5 µM D151N failed to deform membranes (Fig 5A, Fig. S3B). However, mixing 500 nM WT N-BAR with 4.5 µM D151N mutants (achieving identical *total* protein concentration as in Figs. 4&5) successfully rescued the N-BAR tubulation ability (Fig. S3A&B). Both budding and long tubule formation (>1 µM) were found in such samples (Fig. S3A&B). This observation supports our hypothesis that the defect for D151N tubule generation lies in impaired spontaneous curvature initiation (budding), instead of lack in cylindrical curvature stabilization.

**Figure 8:**
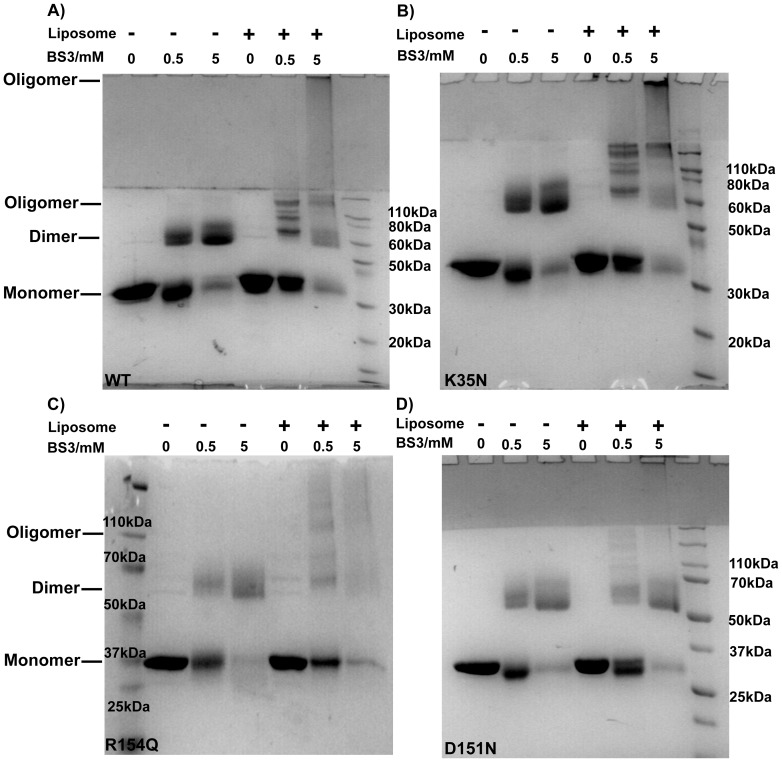
Chemical crosslinking reveals that D151N mutation impairs protein oligomerization upon membrane binding. A) WT, B) K35N, C) R154Q and D) D151N are incubated in absence or presence of 100% DOPS LUVs (0.1 mg/mL, final concentration) at room temperature for 30 mins. Indicated amount of BS3 (Bis[sulfosuccinimidyl] suberate) is added in each sample followed by incubation at 37°C for 2 min. Samples are analyzed by SDS-PAGE gel and stained by Coomassie staining. In absence of liposome, N-BAR domain proteins are crosslinked into dimer with MW around 66 kDa. In presence of liposome, WT and K35N show oligomers bands at 0.5 mM BS3 concentration. Further increasing crosslinker concentration yields species which cannot enter the resolving gel. R154Q in C) shows weaker crosslinked bands while D151N in D) shows major dimer bands in presence of membrane. The higher molecular weight band is absent even at 5 mM BS3 concentration in the D151N sample. Buffer: 20 mM Hepes, 150 mM NaCl, pH 7.4.

Cryo-EM reconstructions and simulations of endophilin N-BAR revealed that oligomeric assembly on flat and tubular membranes is essential for inducing and stabilizing tubulation[35], [36]. Additionally, recent computer simulations have proposed that transient protein lattice formation/aggregation on flat membranes is a prerequisite for tubulation induced by the N-BAR domain[62], [63]. Here, we used a chemical cross-linking assay to ask the question if failure in membrane budding by the D151N mutant was due to defective protein oligomerization[64]. In the absence of crosslinkers, BIN1 N-BAR and variants were monomeric in solution under denaturing conditions. SDS-PAGE gels showed a single band corresponding to a molecular weight around 30 kDa. At 0.5 mM or 5 mM crosslinker concentration, BIN N-BAR domains were crosslinked into dimers in solution (Fig. 8). However, when liposomes were present, multiple oligomeric bands with molecular weight larger than a single dimer appeared on SDS-PAGE gels for the cases of WT and K35N, demonstrating that higher-ordered protein complexes were formed when associated with membranes (Fig. 8A&B). For these two proteins, after adding 5 mM BS3, a fraction of the crosslinked species became too large to be able to enter the resolving gel. Addition of BS3 crosslinker to the mixture of R154Q and liposomes resulted in diminishing dimer bands and an unresolvable pattern of oligomeric species on the gel. The unresolvable oligomer pattern of R154Q could be a result of the lowest membrane association affinity among all mutants. In contrast, we observed only dimer bands for D151N mutants in the presence (or absence) of liposomes confirming that the capacity to form protein assemblies on the membrane is impaired for the D151N mutant. Even at high crosslinker concentration (5 mM), absence of protein retention in the stacking gel indicated absence of D151N oligomers (Fig. 8D).

BAR domain protein assemblies are proposed to be stabilized by amphipathic helices as in endophilin N-BAR domains[35], [36], or through edge-edge interactions such as in various F-BAR domains[36], [65], [66]. The intermolecular contacts in F-BAR domains are generated through charged residues at the contact interfaces[65], [66]. Since residue D151 is located in the arm region with the side chain pointing outward in the crystal structure, this orientation might allow for interaction with a charged residue from a neighboring BAR domain in an anti-parallel manner. The crystal structure reveals several charged or polar residues at the distal tip region. We screened these residues by mutation to alanines and transfected the resulting mutants in C2C12 cells to determine if they were important for maintenance of tubulation capacity as well.

**Figure 9:**
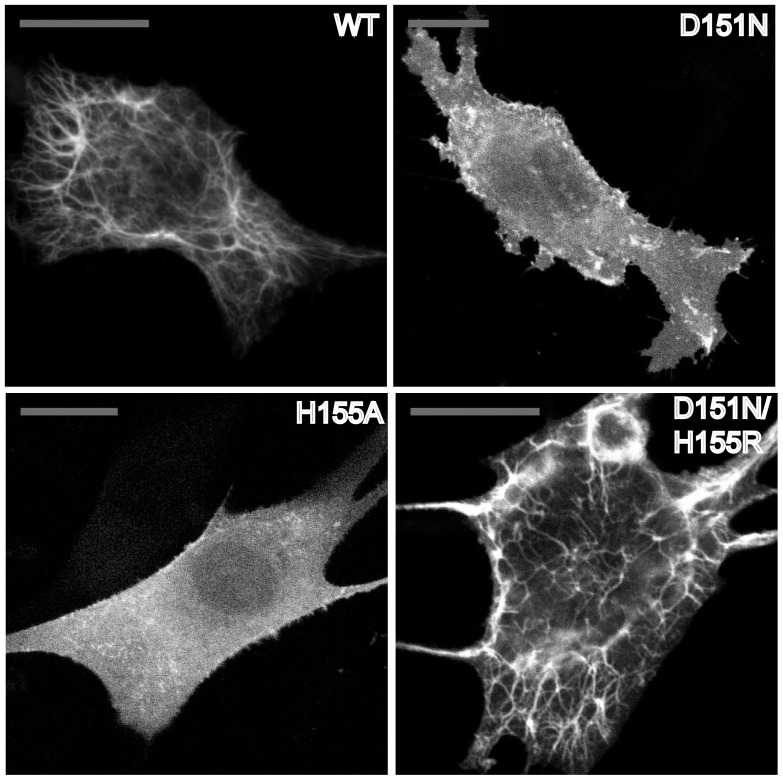
D151N/H155R double mutant rescues tubulation ability in C2C12 cells. BIN N-BAR* WT and its mutants are fused to GFP and transiently transfected in C2C12 myoblasts. Cells are imaged by confocal fluorescence microscopy. Scale bar: 20 µm. Expression of BIN1 N-BAR* WT causes great membrane tubulation in C2C12 cells. H155 is identified as a key residue in regulating N-BAR* domain tubulation ability. Mutating H155 to alanine abolishes tubulation in C2C12 cells while double mutant-D151N/H155R deforms membrane into tubular structures.

After a series of mutational analyses (confer Table 1), residue H155 emerged as a possible candidate interacting with D151. H155A resulted in loss of membrane tubulation when expressing the GFP-conjugated form in C2C12 myoblasts similarly to the D151N mutant (Fig. 9). Our results imply that the conservation of charge either at the 151(-) or 155(+) position is important for maintaining tubulation ability of the BIN1 N-BAR domain (confer Table 1). Additionally, we observed that a double mutation D151N/H155R rescued membrane tubulation in cells. Membrane invaginations marked by green fluorescence in C2C12 cells appeared with tubule morphology similar to the WT (Fig. 9).

**Table 1:** Mutational screening of residues involved in inter-molecular oligomerization at the distal arm of BIN1 N-BAR domain.

Mutants	Tubulation Capacity in transfected C2C12 cells	Mutants	Tubulation Capacity in transfected C2C12 cells
D151E	+++	R154E/E158R	/
R154K	+++	R154Q/E158D	/
H155A	/	R154Q/Y150D	/
H155R	+++	R154Q/Y157D	/
D151N/H155R	+++	R154Y/Y157R	/
		R154Q/Y157R	/

+++ **Tubulation capacity** ***in vivo*** **is comparable to WT BIN1 N-BAR.**

**/No tubulation in cells.**

### Tubulation induced by BIN1 N-BAR domain is antagonized by actin polymerization

**Figure 10:**
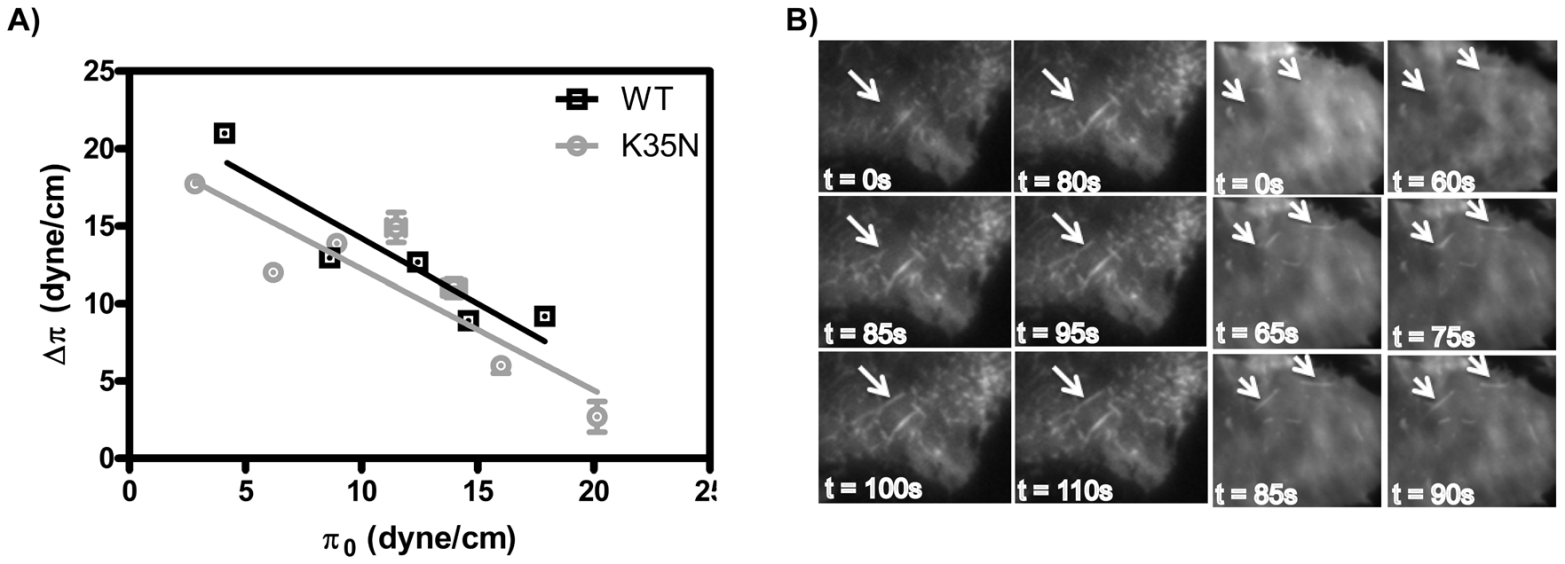
K35N does not change membrane insertion and depolymerizing actin in cells allows membrane deformation. A) WT and K35N BAR domain insertion was studied by monolayer composed of DOPC/DOPS/PI (4,5)P_2_/DOPE (60∶20∶10∶10). π_c_ was determined by extrapolating the Δπ versus π_0_ plot to the abscissa. π_c_ is 26.2 and 25.6 dyne/cm for WT and K35N respectively. Error bars represent standard error of the mean from three independent experiments. An *F*-test of the fittings indicates two linear regressions are not significantly different. B) TIRF images of C2C12 myoblasts transfected BIN1 N-BAR* WT or K35N maintained at 37°C. At t = 0 s, cell culture medium containing 1 µM Latrunculin A are added into the culture dish and time-lapse images are taken. New membrane tubules are generated after actin is depolymerized as the arrows indicate. No increased tubulation was observed in cells transfected with D151N and R154Q N-BAR* (data not shown).

Thus far, we have not been able to identify any conditions under which K35N can be distinguished from WT BIN1 N-BAR domains except in cellular studies. We first recall that the K35 residue is predicted to be projected onto the charged surface of the N-terminal helix in BIN1[17], thus the K35N mutation may influence helix insertion into the lipid membrane and influence membrane tubulation. To test this, we compared the interaction of WT and K35N BAR domain with lipid monolayers[44], [67]. The lipid monolayer was spread at a constant area at a given initial surface pressure (π_0_), and the change in surface pressure (Δπ) was monitored on a Langmuir trough after injection of proteins into the subphase. A linear relationship between Δπ and π_0_ was observed that allows determination of the critical penetration pressure π_c_, which can be interpreted as the upper limit of π_0_ that allows protein penetration into the lipid membrane. We determined close π_c_ values for the WT and K35N BAR domains: 26.2 and 25.6 dyne/cm. According to an *F*-test on the linear regressions in Fig. 10A, the slopes are not significantly different but the intercepts (π_c_) differ slightly (p = 0.024). However, the π_c_ values we determined here are below 31 dyne/cm, which is an estimated surface pressure for the cell membrane[44]. Thus the N-terminal helix insertion may not be the key reason for the reduced membrane deformation capacity by K35N mutant in the *in-vivo* study.

Furthermore, it has been reported that an extended N-BAR peptide that carries the N35 mutation cannot be distinguished from the 1–34 peptide in biophysical measurements including a tubulation assay[68]. Thus, we next asked if there are other cellular processes regulating membrane tubulation and if perturbation of such processes might reveal a role of the K35N mutation. Specifically, we tested the role of actin in the membrane tubulation process and asked how perturbation of actin polymerization affects tubulation by disease mutants.

To that end, C2C12 myoblasts were transfected with GFP-labeled BIN1 WT and K35N N-BAR* domains, and examined using TIRF microscopy. At *t* = 0 s, cells were treated with. 1 µM Latrunculin A, an actin polymerization inhibitor. GFP-positive membrane tubules started to grow from the plasma membrane shortly after inhibitor addition in cells expressing BIN1 N-BAR domains, or alternatively, the K35N mutant (Fig. 10B), similarly as reported before both for F-BAR domain proteins and other N-BAR proteins[69], [70]. Importantly, we did not observe tubule formation from cells expressing D151N and R154Q mutants after depolymerizing the actin cytoskeleton (data not shown). This is consistent with our findings that D151N and R154Q are mutations that cause inability to deform membranes, while K35N behaves similarly to WT. This suggests that the tubulation ability of the K35N mutant is inhibited in cells, possibly by the cytoskeleton. However, it remains unknown if there are other proteins involved in coupling BIN1 to cytoskeletal components, and how the mutated N-terminal helix is involved in this inhibitory interaction.

**Figure 11:**
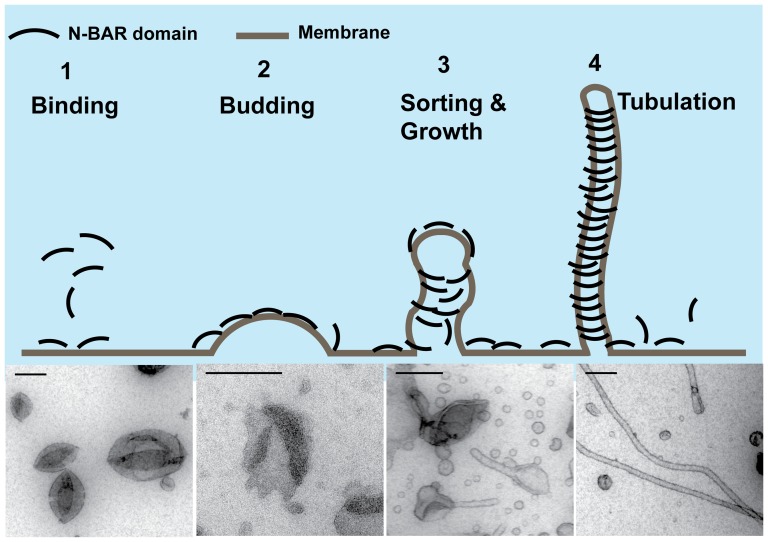
Proposed schematic illustration of BIN1 N-BAR domain tubulating membrane. 1) N-BAR domains bind on membrane; 2) Proteins oligomerize locally to create membrane buds; 3) N-BAR domains sense local curvature and diffuse onto the tubule to further elongate membrane tubule and stabilize the highly curved membrane; 4) Finally, the tubules are fully decorated by N-BAR domains. Electron micrographs are N-BAR domain WT (200 nM–5 µM) incubated with DOPC/DOPS/PI (4,5)P_2_/DOPE (60∶20∶10∶10) at room temperature. Scale bar: 200 nM.

In conclusion, our studies have shown that both protein density and oligomerization on membranes determine membrane curvature generation ability. Based on the membrane structures revealed in the tubulation assay, we suggest that in order to initiate spontaneous liposome deformation and tubule growth, transient ordered protein oligomers are required to form on a flat membrane and to allow for the initiation of tubule formation. A schematic illustration of this process is shown in Fig. 11. We have divided the tubulation process into the following four steps:

1. A membrane association step enables proteins to reach a certain density on the membrane. In the class of N-BAR domains, this interaction is primarily mediated by electrostatic interactions between charged residues located at the binding interface with negatively charged lipid headgroups. The membrane insertion of the amphipathic helix in the BAR domain also contributes to increasing the protein density on the membrane. GUV isotherm measurements demonstrated that the R154Q mutation led to decreased protein density on GUVs. We hypothesize that the reduced binding may result from the unfavorable charge change at residue 154, where a highly membrane binding and inserting residue has been mutated to a neutral one. Although, based on the crystal structure, R154 is located in the tip region of the BAR domain and the orientation of this residue does not point directly to the membrane binding interface. Given the length and the flexibility of the Arginine side chain, we hypothesize that the R154 residue may adopt a conformation allowing interactions with anionic lipids, thus the mutant R154Q may influence membrane binding by interrupting electrostatic interactions.
2. With increasing protein density on the membrane, local curvature is induced as budding sites for tubule formation grow; a process where protein oligomerization is involved[62]. At an intermediate protein concentration (500 nM), liposome boundaries developed wobbles and semi-spherical structures appeared on the membrane. We consider those buds as the initiation sites for tubulation to occur. In our experiments, we observed bud formation for both WT and K35N mutants, and for R154Q mutants at higher protein concentration. However, budding formation was absent in D151N mutants even at 15 µM concentration. Our chemical crosslinking assay further confirmed that covalently crosslinked protein oligomers occurred under conditions where membrane deformations were induced by N-BAR domains. The D151N mutant specifically abolished protein assembly on the membrane. Our observations suggest a correlation between protein nucleation and membrane bud formation. This hypothesis is further supported by recent computational simulations, which reported that N-BAR domains form linear aggregates on the membrane, with emerging membrane buds at low surface protein densities[35], [62], [63].
3. After the initial curved membrane is formed, BAR domains can sense the high curvature membrane and migrate onto tubules to facilitate tubule growth. Currently, the most popular interpretation of the membrane curvature sensing mechanism is that it is mediated by N-terminal amphipathic helix insertion in which curved membranes display increased membrane defect density promoting hydrophobic insertion[4], [41], [50], [51]. In our protein partitioning assay, we did not observe significant differences in curvature sensing ability among BIN1 N-BAR mutants. For the D151N and R154Q mutants, the intact N-terminal helix likely provides a driving force for curvature sensing. Strikingly, we observed that the K35N behaved similarly to WT BIN1 N-BAR domain. Previous studies on an extended N-terminal peptide of human Amphiphysin II (residue 1–44) have shown that K35N mutants cannot be distinguished from WT peptide in CD/NMR spectra and tubulation assays[68]. Together with our *in vitro* biophysical measurements, it can therefore be inferred that the K35 residue is not critical for amphipathic helix function. Our preliminary findings implicate that the cytoskeleton plays a role, because upon actin depolymerization, K35N mutant in cells are able to tubulate membranes just as WT N-BAR. However, future work is needed to understand the role of the K35 residue in regulating protein tubulation ability.
4. More proteins are sorted onto the tubular membrane and a membrane tubule becomes decorated by a protein coat to stabilize the generated curvature indicated in previous EM studies.

We have experimentally shown that protein-protein assembly is required to drive membrane bud formation in the early stage of membrane deformation which is consistent with the results from simulations[63]. However, these simulations did not provide all-atom resolution to reveal the residues involved in lateral contacts among neighboring N-BAR domains. A mutagenesis screening performed at the BIN1 N-BAR tip region allowed us to identify potential residues regulating membrane deformation capacity. As a result, H155 caught our attention because mutation of this residue to a neutral amino acid abolishes tubule formation by N-BAR domains *in-vivo*. The fact that the double mutant D151N/H155R rescues tubulation ability of the BAR domain in cells is surprising. As expected, the purified double mutant retained the *in-vitro* membrane deformation capacity and membrane-mediated oligomerization (data not shown). One possible explanation is that D151 and H155 engage in an inter-molecular interaction of two BAR domain dimers on the membrane and facilitate the formation of a protein lattice. This might imply that arginine at the 155 position enhances the inter-molecular H-bond strength and may thus rescue the tubulation capacity in the disease mutant. This may be due to the fact that the enthalpic gain upon formation of the charged-neutral H-bond (D151…H155 in WT or N151…R155 in the double mutant) is greater compared to a neutral-neutral H-bond (N151…H155 in the disease mutant)[71]–[73]. To further test this idea, we constructed a double mutant where these two residues were swapped, and we expressed it in C2C12 myoblasts. Unfortunately, this test proved unsuccessful due to protein aggregations into vacuole-like structures. Future studies such as high-resolution reconstruction from Cryo-EM images may provide direct evidence for potential contact formation of these two residues upon membrane binding.

In this study, we have focused on the differences in the membrane deformation ability among disease-related mutations in the N-BAR domain of BIN1. We hasten to remark that T-tubule biogenesis in skeletal muscle tissues is likely to be a more complicated process than discussed here. This is in part due to the fact that in the full-length BIN1 protein, exon10-encoded peptide and SH3 domain contribute to the regulation of membrane deformation ability of the BIN1 protein. Particularly, the SH3 domain acts as an adaptor to allow BIN1 complexation with other proteins to modulate membrane morphology. In addition to dynamin2, it has been recently discovered that myotubularin (MTM1) binds the BIN1 SH3 domain and enhances BIN1-mediated membrane tubulation. Binding between SH3 domain and downstream proteins such as dynamin2 and MTM1 induces a conformational change in full-length BIN1 that favors membrane deformation[74]. This finding supports a more complex pathological mechanism in centronuclear myopathy. The mutations we examined in this study do not have an impact on MTM1 recruitment to BIN1 because they were located outside of the SH3 domain. This finding also supported our argument that mutations in the N-BAR domain did not alter the protein conformation as confirmed by CD spectroscopy and size exclusion chromatography. Thus, the biophysical measurements we performed here provide a basis for elucidating molecular mechanisms contributing to membrane deformation defects in the CNM-related mutations, specifically in the N-BAR domain.

## Materials and Methods

1,2-dioleoyl-sn-glycero-3-phosphocholine (DOPC), 1,2-dioleoyl-sn-glycero-3-phospho-L-serine (DOPS), 1,2-dioleoyl-sn-glycero-3-phosphoethanolamine (DOPE), L-α-phosphatidylinositol-4,5-bisphosphate(Brain), distearoylphosphatidylethanolamine-N-(biotinyl(polyethylene glycol)2000) (DSPE-Bio-PEG2000), and cholesterol were purchased from Avanti Polar Lipids (Alabaster, AL). Alexa Fluor 488 C5-maleimide and TexasRed-1, 2-dihexadecanoyl-sn-glycero-3-phos-phoethanolamine triethylammonium salt (TR-DHPE) were from Invitrogen (Carlsbad, CA). Streptavidin-conjugated microspheres with a diameter of 6 µm were from Polysciences (Warrington, PA).

### DNA constructs expression and purification of recombinant proteins

BIN1 N-BAR* (1–282) in pEGFP-C2 vector and GST-fused BIN1 N-BAR (1–254) in pGEX-4T-2 were kindly provided by the De Camilli lab. DNA sequence coding BIN1 N-BAR* (1–282) was amplified by PCR and inserted into the mKate-N1 vector. CNM-associated mutations were introduced by standard primer-directed PCR mutagenesis. All constructs were confirmed by sequencing. GST-fusion proteins of BIN1 and its variants were expressed in BL21-Codon Plus (DE3)-RIL bacteria (Stratagene). Cells were grown at 37°C to OD600 of 0.8 and induced with 1 mM IPTG for 3 hours at 37°C. Then they were harvested by centrifugation, resuspended in lysis buffer (20 mM Tris-HCl, 300 mM NaCl, 1 mM DTT, 1 mM PMSF, pH 7.4) and lysed on ice by tip sonication. After centrifugation, the supernatant was applied to a GST-affinity column equilibrated with the lysis buffer and eluted with elution buffer (50 mM Tris-HCl, 20 mM reduced glutathione, pH 8.0). The GST-tag was cleaved via thrombin digestion at room temperature for 5 hours and the untagged BIN1 N-BAR proteins were further purified by cation exchange and gel filtration (GE Healthcare). The endogenous cysteine residues were labeled by Alexa 488 C5-maleimide (Invitrogen) at 4°C overnight. Free fluorophores were removed via desalting column three times. Protein concentrations were measured by standard Bradford Assay (Thermo Scientific) triple times (with 0.5%–1% uncertainty in protein concentration determination) and concentrations of fluorophores were determined by absorbance at 494 nm (with molar extinction coefficient 71,000 cm^−1^M^−1^). Labeling efficiency was calculated by: Labeling efficiency [%] =  Alexa 488 concentration/Protein concentration * 100. All the protein samples used in our studies were freshly thawed and ultra-centrifuged to remove potential aggregates. Protein concentration was determined by Bradford assay before each experiment. No sample stored at 4°C for longer than one week was used in this study.

### Cell culture, transfection and confocal fluorescence imaging

C2C12 myoblasts were cultured in DMEM medium containing 10% fetal bovine serum (FBS) (Invitrogen). Cells were cultured in MatTek glass bottom culture dishes until 95% confluency before transfection. 1.5 µg EGFP-tagged or mKate-tagged DNA were transfected with Lipofectamin2000 (Life Technologies, Invitrogen) and incubated at 37°C, 5% CO_2_ for 5 hours before changing to culture medium. Cells were imaged after 24 hours with a fluorescence confocal microscopy (FV300) scanning system integrated with a motorized inverted microscope IX81 (Olympus, Center Valley, PA) using a 60 x, 1.2 NA water immersion lens (Olympus). In order to investigate the spatial distribution of the N-BAR* domain of BIN1 on plasma membranes, TIRF imaging was performed on an inverted IX71 microscope system equipped with a 60 x, 1.45 NA TIRF objective (Olympus) using 50 mW 488 nm laser (Coherent, Santa Clara, CA) and appropriate neutral density filters (Thorlabs, Newton). Images were imported into and processed with ImageJ.

### Liposome preparation

Liposomes were prepared using either 100% DOPS, or 60%DOPC/20%DOPS/10%PI(4,5)P_2_/10% DOPE. Lipids were mixed and air-dried to form lipid films and rehydrated by 20 mM Hepes, 150 mM NaCl, 1 mM DTT, pH 7.4 buffer with final concentration 1 mg/mL. Liposome solutions were sonicated for 15 minutes and extruded through 400 nm nuclepore membranes (Whatman) 11 times. All liposome solutions were stored at 4°C.

GUVs (100%DOPS and 60%DOPC/20%DOPS/10%PI(4,5)P_2_/10%DOPE) were prepared by electroswelling method in 0.3 M sucrose solution. The osmolarity of formed GUV dispersion was measured by a micro-osmometer (Advanced Instruments Inc., Norwood, MA).

### Liposome tubulation assay

5 µM BIN1 N-BAR proteins were incubated with liposomes (100% DOPS or 60% DOPC/20% DOPS/10% DOPE/10% PI(4,5)P_2_, 0.1 mg/mL) in 20 mM Hepes, 150 mM NaCl, 1 mM DTT, pH 7.4 buffer at room temperature for 30 minutes. Samples were absorbed on carbon/formvar supported copper grids (Electron microscopy science, Hatfield, PA) for 1 minute and excess samples were washed by blotting on a filter paper (Whatman). The grids were then stained with 2% (w/v) uranyl acetate for 1 minute, washed, and dried under room temperature. Grids were observed with a JEM 1011 transmission electron microscope (JEOL, USA) with the accelerating voltage set to 100 kV. Images were analyzed by ImageJ.

### Protein densities quantifications on GUV membrane and Langmuir isotherm

DOPC mixed with 0.05%∼0.7% BODIPY-DHPE were made via electroformation method and imaged with confocal microscopy. The mean grey level on a GUV equator was measured by ImageJ and plotted against BODIPY concentration. BODIPY densities were estimated by using averaged lipid headgroup size as 0.72 nm^2^. To figure out the ratio of fluorescence intensities between BIN1 N-BAR-Alexa488 and BODIPY-DHPE, we measured the bulk fluorescence of SUVs (50 nm in diameter) containing 0.05%∼0.7% BODIPY-DHPE and BIN1 N-BAR-Alexa488 as a function of fluorophore concentration. The ratio of the linear fitting slope corresponds to a relative efficiency of Alexa 488 to BODIPY-DHPE[45]–[47]. 11 µM GUVs were incubated in 20 mM Hepes, 50 mM NaCl, pH 7.4 buffer containing increasing concentrations of BIN1 N-BAR* or its mutants. Samples were incubated at room temperature for 30 mins before imaging by confocal microscopy. Images were imported into ImageJ and the density of bound proteins on the GUV was measured as the mean grey level on a GUV equator and converted to molecular densities based on an adapted fluorescence calibration method described above.

### Tether pulling force and membrane lateral tension measurements

Micropipettes were fashioned from glass capillaries (World Precision Instruments Inc., Sarasota, FL) that were stretched using a pipette puller. Pipette tips were cut using a microforge at desired inner diameters of 5∼8 µm. Irreversible adhesion of membrane to the pipette was prevented by filling the pipette tips with 2.5 mg/ml casein dissolved in 1x PBS. Pipettes were then rinsed and filled with 300 mM sucrose solution using a MicroFil needle (WPI, Sarasota, FL). A sample chamber was formed from two coverslips overhanging both sides of a microscope glass slide, creating a 1 mm thick cell that was open on three sides to allow the insertion of a micropipette. The bottom of the chamber was pre-coated with 2 µL of 2.5 mg/ml casein (dissolved in 1x PBS) to prevent the adhesion of microspheres and GUVs to the coverslip. A mixture of sucrose and buffer solution with the same osmolarity as the GUV dispersion was prepared while keeping the NaCl concentration at 50 mM. The chamber was filled with 90∼100 µL of sucrose and buffer mixture, 1 µL of microsphere dispersion and 3 µL of GUV dispersion. BIN1 was added to reach the desired concentration. The chamber was mounted on an inverted microscope (1×71; Olympus, Center Valley, PA) equipped with a home built optical trap, which uses a second, independently positioned objective (60X, 1.1 NA, water immersion, 1.5 mm working distance; Olympus) oriented opposite to the imaging objective to introduce a 1064 nm wavelength laser into the chamber. Micropipettes were moved via a three-dimensional motorized micromanipulator system (Luigs & Neumann, Ratingen, Germany). Aspiration pressure was controlled through adjustments of the height of a water reservoir, and pressures were measured with a differential pressure transducer (Validyne Engineering, Los Angeles, CA). The chamber was equilibrated for 10∼20 min before an individual GUV (typically between 8 and 15 µm in radius) was aspirated at a constant pressure. A subsequently trapped bead was brought into contact with the vesicle, and then either the vesicle or the bead was retracted at a speed about 5 µm/s, generating a membrane tether around 10∼20 µm in length. Forces exerted on the bead were measured in real-time. Aspiration pressure was changed subsequently to generate the relation between tether pulling force and membrane lateral tension. Each lateral tension was maintained until the pulling forces reached equilibrium (typically 1∼2 min).

### Optical trap Design and Calibration

Our home-built optical trap has been previously described[54]. The pulling force *f* exerted by the tether is determined as for a Hookean spring: *f* =  *k*Δ*x*, where *k* is the trap stiffness and Δ*x* is the displacement of the bead relative to its equilibrium position. The stiffness of the trap was calibrated by the drag-force method for at least 3 beads before the tether pulling experiment and was typically 0.05 pN/nm. After each experiment, the trap stiffness was recalibrated to confirm the previously measured value and monitor the possible influence of chamber solution evaporation on the measured stiffness of the optical trap.

### BIN1 WT and mutants curvature sorting measurements

To investigate curvature-sensing abilities of BIN1 WT and mutants, we examined their fluorescence intensities on the tubular membrane under varying membrane tensions. The experiments were carried out in a chamber constructed from glass slides, containing GUV dispersions and protein solution. Two micropipettes (World Precision Instruments, Sarasota, FL), which were fabricated by using a microforge, were inserted into the sample chamber by a three-dimensional motorized manipulator system (Luigs and Neumann, Ratingen, Germany). Vesicles in the chamber were aspirated via micropipette. The membrane tension of the vesicle was controlled by adjusting the height of a connected water reservoir, and measured by a pressure transducer with a DP-28 diaphragm (Validyne Engineering, Los Angeles, CA). The other pipette then aspirated a bead, contacted the vesicle, and was moved away to pull a membrane tether. We used Kalman-averaged images of the tether cross section (xz plane, which was orthogonal to the axis of the tether) for measuring tether fluorescence intensities, with a stepwidth of 0.15 µm and a total imaging depth of 6 µm. Such recorded tether fluorescence intensity profiles were then background-corrected, and estimated in an elliptical region of interest to obtain the protein and lipid intensity signals under varying tensions. For each protein variant, at least five independent experiments were executed, analyzed and binned to obtain the final result.

### Chemical cross-linking assay

For BIN1 N-BAR variants crosslinking experiments, 6.6 µg (5 µM) BIN1 N-BAR protein and CNM-related mutants were mixed with 100% DOPS liposomes (0.1 mg/mL) in a total volume of 40 µl of 20 mM Hepes, 150 mM NaCl, pH 7.4 buffer. All samples were incubated for 30 mins at room temperature before adding cross-linking reagent bissulfosuccinimidyl suberate (BS3) (Thermo Scientific) from a 100 mM stock in distilled water to reach final concentrations of 0, 0.5, and 5 mM. Samples were further incubated for 2 min at 37°C. Laemmli sample buffer was added to terminate the reactions. Samples were boiled at 95°C for 5 min, subjected to SDS-PAGE electrophoresis and visualized by Coomassie staining. Control samples were prepared by replacing liposomes with incubation buffer.

### Actin depolymerization in BIN1 N-BAR* variant transfected cells

C2C12 myoblasts were transfected with BIN1 N-BAR* variants tagged with EGFP in Bioptechs delta T culture dishes. Cells were maintained at 37°C by Bioptechs objective and culture dish heaters (Fisher). Images were collected by a 60 x, 1.45 NA TIRF lens (Olympus, Center Valley, PA) on an inverted microscope system (IX71, Olympus, Center Valley, PA) using a 488 nm laser (50 mW, Coherent, Santa Clara, CA). 1 µM latrunculin A (Biomol International, Plymouth Meeting, PA) containing culture medium was added into culture dish. Data were recorded every 100 ms using a cooled EMCCD camera (HAMAMATSU, Bridgewater, NJ).

### Monolayer insertion measurements

Insertion of WT and K35N N-BAR domains into a lipid monolayer was investigated by measuring a change in the surface pressure (π) at constant surface area using a 1 mL circular Teflon trough and wire probe connected to a Kibron MicroTrough X (Kibron, Inc., Helsinki). A lipid monolayer containing 60% DOPC, 20% DOPS, 10% PI(4,5)P_2_ and 10% DOPE was spread onto the sub-phase composed of 20 mM Hepes, 150 mM NaCl at pH 7.4 until the desired initial surface pressure (π_0_) was reached. Then excess N-BAR protein was injected into the sub-phase through a hole in the wall of the trough. The change in surface pressure (Δπ) was monitored after equilibration. The resulting Δπ was plotted versus π_0_, and critical surface pressure (π_c_) was determined as the x-intercept.

## Supporting Information

Figure S1 Expression of EGFP or mKate alone in C2C12 cells does not induce plasma membrane tubulation. Confocal images of C2C12 cells transfected with A) pEGFP-N1 vector; B) mKate-N1 vector. Scale bar: 20 µm.
(TIF)

Figure S2 Position of fluorescence tag does not change tubulation abilities of BIN1 N-BAR* variants. A) BIN N-BAR* WT and mutants are fused to EGFP at the N-terminus and transiently transfected in C2C12 myoblasts. Cells are imaged via confocal fluorescence microscopy. Similar tubulation phenotypes are observed as in the case of the C-terminal labeled proteins. Scale bar: 20 µm. B) Quantification of three types of membrane deformations by BIN1 N-BAR* variants. Clusters are defined as structures with lengths not longer than five times of their width. Over 50 cells were analyzed in each separated experiments. Error bars: standard error of the mean in black and standard deviation in light grey. Student *t*-test for statistical significance: n.s: p>0.05, *: p<0.05, **: p<0.01, ***: P<0.001.
(TIF)

Figure S3 Mixing D151N mutants with 500 nM WT N-BAR restores tubulation. Electron micrographs of liposome A) 500 nM WT+4.5 µM D151N. Scale bar: 200 nm. B) Quantification of tubulation enhancement after mixing WT N-BAR with D151N. 500 nM WT primarily generates membrane buds on vesicles with occasional tubules found on the grid. D151N consistently had a negligible effect on membrane morphology. When mixing D151N N-BAR with 500 nM WT N-BAR domain, tubulation was observed suggesting D151N was able to induce membrane tubule elongation as long as the initiation intermediates were present. Over 30 images were analyzed. Error bars: standard error of the mean in black and standard deviation in light grey. Student *t*-test for statistical significance: n.s: p>0.05, *: p<0.05, **: p<0.01, ***: P<0.001.
(TIF)
