# Supplementary figures and images for: Mutations in BIN1 Associated with Centronuclear Myopathy Disrupt Membrane Remodeling by Affecting Protein Density and Oligomerization
Source: PLoS One. 2014 Apr 22;9(4):e93060. doi: 10.1371/journal.pone.0093060 (PMC3995651; doi:10.1371/journal.pone.0093060)

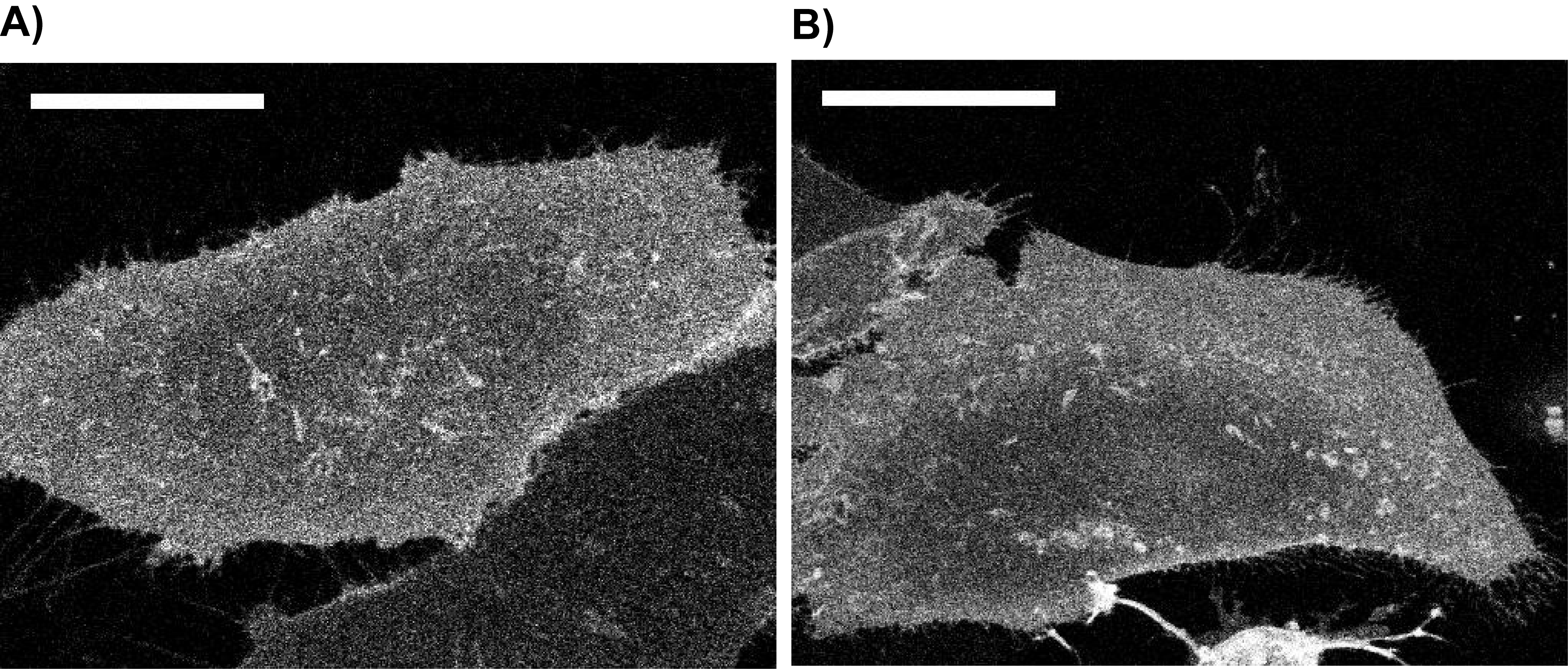

Supplement: Figure S1 — Expression of EGFP or mKate alone in C2C12 cells does not induce plasma membrane tubulation. Confocal images of C2C12 cells transfected with A) pEGFP-N1 vector; B) mKate-N1 vector. Scale bar: 20 µm. (TIF) [file pone.0093060.s001.tif]

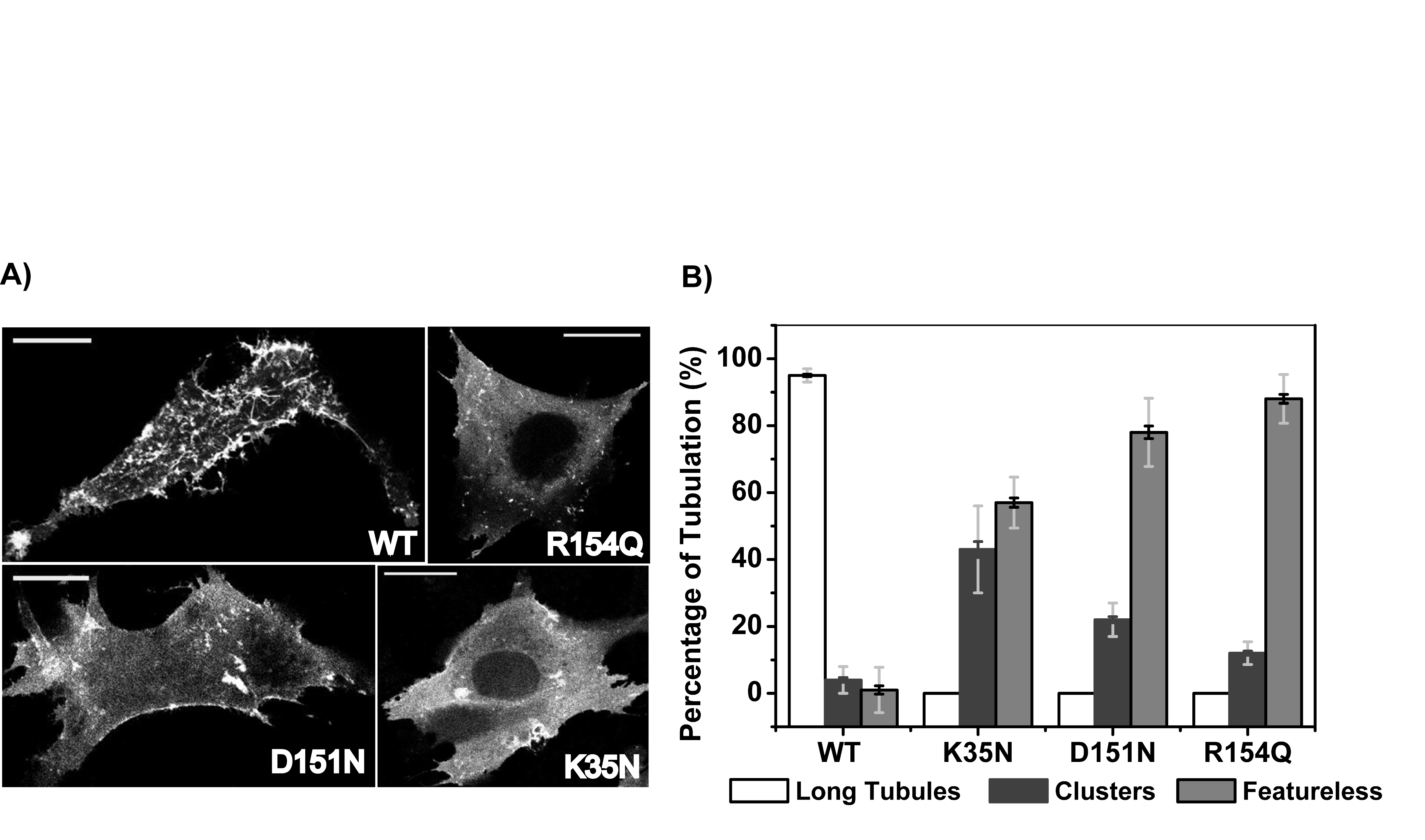

Supplement: Figure S2 — Position of fluorescence tag does not change tubulation abilities of BIN1 N-BAR* variants. A) BIN N-BAR* WT and mutants are fused to EGFP at the N-terminus and transiently transfected in C2C12 myoblasts. Cells are imaged via confocal fluorescence microscopy. Similar tubulation phenotypes are observed as in the case of the C-terminal labeled proteins. Scale bar: 20 µm. B) Quantification of three types of membrane deformations by BIN1 N-BAR* variants. Clusters are defined as structures with lengths not longer than five times of their width. Over 50 cells were analyzed in each separated experiments. Error bars: standard error of the mean in black and standard deviation in light grey. Student t-test for statistical significance: n.s: p>0.05, *: p<0.05, **: p<0.01, ***: P<0.001. (TIF) [file pone.0093060.s002.tif]

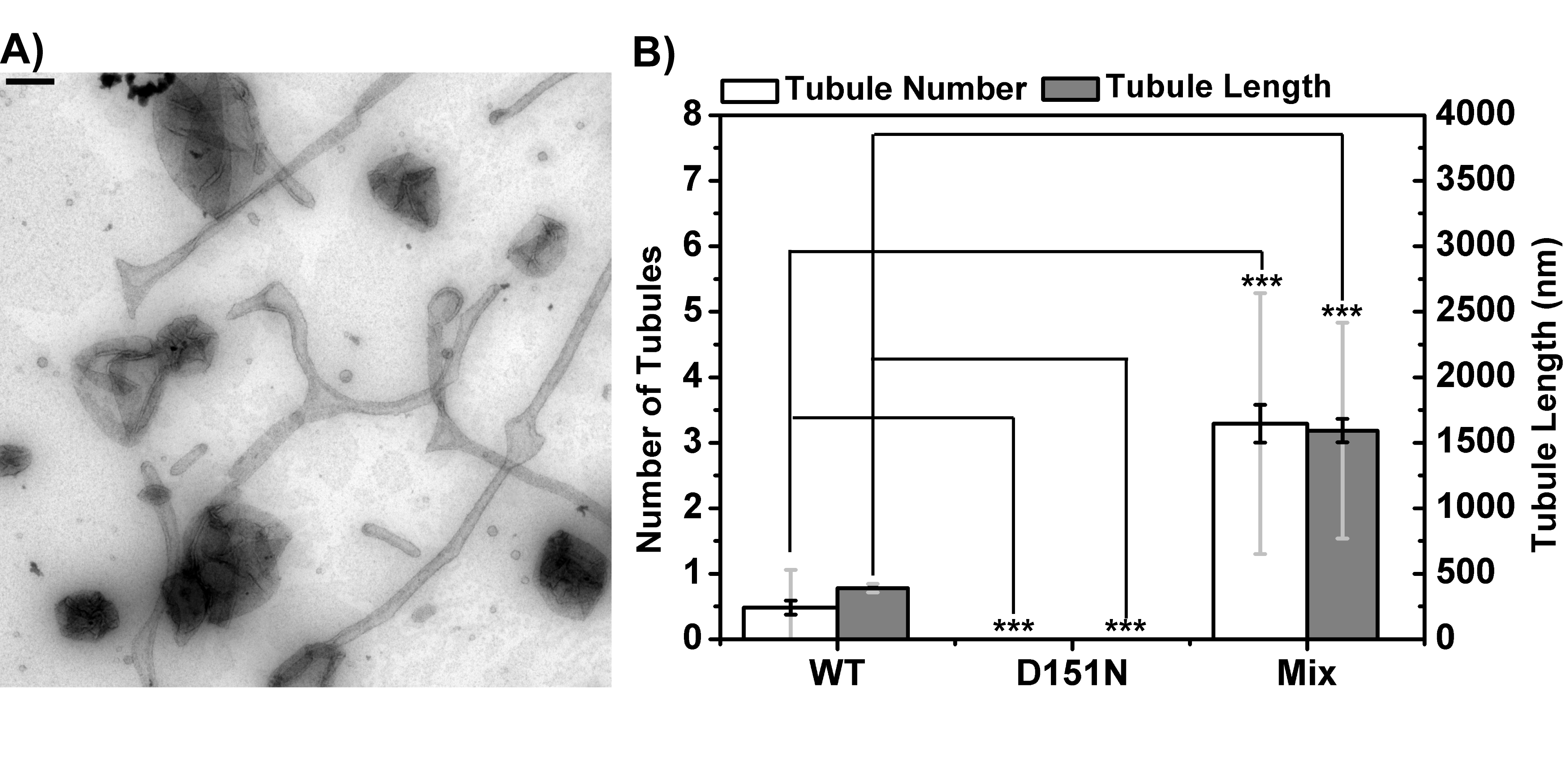

Supplement: Figure S3 — Mixing D151N mutants with 500 nM WT N-BAR restores tubulation. Electron micrographs of liposome A) 500 nM WT+4.5 µM D151N. Scale bar: 200 nm. B) Quantification of tubulation enhancement after mixing WT N-BAR with D151N. 500 nM WT primarily generates membrane buds on vesicles with occasional tubules found on the grid. D151N consistently had a negligible effect on membrane morphology. When mixing D151N N-BAR with 500 nM WT N-BAR domain, tubulation was observed suggesting D151N was able to induce membrane tubule elongation as long as the initiation intermediates were present. Over 30 images were analyzed. Error bars: standard error of the mean in black and standard deviation in light grey. Student t-test for statistical significance: n.s: p>0.05, *: p<0.05, **: p<0.01, ***: P<0.001. (TIF) [file pone.0093060.s003.tif]
